# Extraction and Characterization of Potential Biodegradable Materials Based on *Dioscorea hispida* Tubers

**DOI:** 10.3390/polym13040584

**Published:** 2021-02-15

**Authors:** K. Z. Hazrati, S. M. Sapuan, M. Y. M. Zuhri, R. Jumaidin

**Affiliations:** 1 Advanced Engineering Materials and Composites Research Centre, Department of Mechanical and Manufacturing Engineering, Universiti Putra Malaysia (UPM), Serdang 43400, Selangor, Malaysia; hazrati88@gmail.com (K.Z.H.); zuhri@upm.edu.my (M.Y.M.Z.); 2 German Malaysian Institute, Jalan Ilmiah, Taman Universiti, Kajang 43000, Selangor, Malaysia; 3 Laboratory of Biocomposite Technology, Institute of Tropical Forestry and Forest Products (INTROP), Universiti Putra Malaysia (UPM), Serdang 43400, Selangor, Malaysia; 4 Fakulti Teknologi Kejuruteraan Mekanikal dan Pembuatan, Universiti Teknikal Malaysia Melaka, Hang Tuah Jaya, Durian Tunggal 76100, Melaka, Malaysia; ridhwan@utem.edu.my

**Keywords:** *Dioscorea hispida* tubers, starch, natural fibres, polymer from renewable source, sustainable materials

## Abstract

This study was driven by the stringent environmental legislation concerning the consumption and utilization of eco-friendly materials. Within this context, this paper aimed to examine the characteristics of starch and fibres from the *Dioscorea hispida* tuber plant to explore their potential as renewable materials. The extraction of the *Dioscorea hispida* starch and *Dioscorea hispida* fibres was carried out and the chemical composition, physical, thermal, morphological properties, and crystallinity were studied. The chemical composition investigations revealed that the *Dioscorea hispida* starch (DHS) has a low moisture t (9.45%) and starch content (37.62%) compared to cassava, corn, sugar palm, and arrowroot starches. Meanwhile, the *Dioscorea hispida* fibres (DHF) are significantly low in hemicellulose (4.36%), cellulose (5.63%), and lignin (2.79%) compared to cassava, corn hull and sugar palm. In this investigation the chemical, physical, morphological and thermal properties of the *Dioscorea hispida* fibre and *Dioscorea hispida* starch were examined by chemical composition investigation, scanning electron microscopy (SEM), particle size distribution, thermogravimetric analysis (TGA), X-ray powder diffraction (XRD), and Fourier transform infrared (FTIR), respectively. It was found that *Dioscorea hispida* waste is promising alternative biomass and sustainable material with excellent potential as a renewable filler material for food packaging applications.

## 1. Introduction

Non-renewable materials have caused critical environmental issues in many countries in the world regarding waste disposal [1,2,3]. This problem is overwhelming for countries with limited landfill resources. A hierarchy of waste handling has been proposed to ensure long-lasting supervision that comprises recycling, reduction in waste, reuse, and landfill [4,5]. The production rate of plastic wastes is very much exceeding the plastic degradation rate, consequently presenting an ecosystem imbalance. This phenomenon could cause waterborne illnesses resulting from water pollution as plastic waste leachate leaks into water supply areas, e.g., artificial and natural lakes, and impoundment developed from a dam or water storage [6,7]. Therefore, to overcome the problem, there is a need to sequentially switch or replace petroleum-based plastics with bioplastics to minimize the reliance upon fossil-based energy as well as to contribute to an easier end of life disposal. The word “bioplastics” arises from the need to build new sustainable systems without renouncing some essential physicochemical characteristics [8,9,10]. This situation has initiated researchers to find alternative materials originating from biofibres and biopolymers [11,12,13,14].

Recent environmental regulation has forced production industries to seek for other potential materials for the development of their product that are more environmentally friendly than synthetic materials (i.e., petroleum-based polymer composites and glass fibres) [15,16]. In recent years, the implementation of agricultural wastes as sustainable filler materials has grown, initiated from the researchers and industrial players. The recognized benefits of these materials are low density, reduced tool wear, and low cost that favour the manufacturing industry [17]. The eligibility of multiple natural waste categories as reinforcements in polymer composites has been realised, e.g., sugar palm [6,18], cassava [19], corn [20], and others. Recent studies have developed the abilities of other fibres to use in polymer composites, such as oil palm, sugarcane, roselle, kenaf, ginger, and bamboo [21,22,23,24,25,26,27]. The advances in implementing the methods used for biomaterial design focus on those derived from formulations based on fruit and vegetable by-products [28,29].

Biocomposites are changing the research activities carried out in the scope of material engineering because the number of beneficial properties proven, such as biodegradability, lightweight, low energy consumption, usability, and eco-friendly [30,31,32,33]. There are some disadvantages associated with these lignocellulosic fibres, along with a variety of advantages as reinforcements. The compatibility between lignocellulosic fibres and other biopolymers has been improved in a large number of studies [9]. This compatibility is impeded by the hydrophilicity and heavy crosslinking of lignocellulosic fibres, resulting in both weak interfacial adhesion and mechanical properties [34]. The dimensional stability of these bioplastics, when exposed to moisture, is limited. Surface modifications are commonly used to improve the efficiency of lignocellulosic fibres and to encourage improved adhesion between the natural reinforcement and the polymeric matrix [35,36,37,38]. By eliminating the complicated lignin, most of the surface modifications are based on cellulose. As a result, lignin is typically used to generate residual waste, while cellulose is used in bioplastics [39]. The high aspect ratio of cellulose fibres (up to 1 μm length), the chemical-modifiable surfaces, and high elasticity properties due to their great crystallinity can be prepared using relatively inexpensive development procedures which offer great potential to produce many functional structures in biocomposite materials based on cellulosic derivatives [40,41].

Currently, biocomposites are used in various applications, e.g., automotive, aircraft, building materials, sports equipment, and food packaging [42]. From the perspective of the packaging industry, starch-based composites products are gaining greater recognition for different packaging applications in the bio-based polymer industry [29,43]. The use of natural fibres as reinforcement in polymer composites provides the composites themselves with more environmentally friendly characteristics [8]. The natural fibres are extracted from plant that composed of chemical constituents like cellulose, hemicellulose, lignin, ash, and wax [44,45]. The mechanical strength, chemical compositions, thermal stability, and crystalline properties of the natural fibres are influenced by the environmental conditions [46,47,48]. Furthermore, other elements affecting the properties of the fibres are the maturity of the plant, method of extraction, and extracted parts of the plant, such as stem, leaf, stalk, and root [47,49]. However, the fraction of the amorphic (lignin, hemicellulose, wax) and crystalline (cellulose) in natural fibres might differ with the location and the condition of the grown plant [50]. The fraction of amorphic content in the fibre influences the properties of the materials, so it is necessary to decrease the amorphic fraction in the natural raw fibres before utilizing them as reinforcements [51].

*Dioscorea hispida* plants, also known as *Ubi Gadong* in Malaysia is a poisonous tuber plant that contains the alkaloid of dioscorine [52]. Recent studies reported over 600 *Dioscorea* sp. discovered in numerous parts around the globe, specifically in the subtropical and tropical regions [53]. Studies have proven that an alkaloid compound from the tubers can cause vomiting, sleepiness in humans, dizziness, and nausea [54]. The starch from *Dioscorea hispida* tubers is edible after the removal of the dioscorine compound [55]. It requires approximately five days of immersing the tuber in distilled water for full detoxification of the dioscorine [56]. The knowledge on the *Dioscorea hispida* starch is largely based upon empirical studies that investigated its use as a crude drug for inflammation [57]. Previous studies of *Dioscorea hispida* starch demonstrated the hydrogel created from the starch functioned as an antibacterial agent to prevent microorganism activity [58]. Additionally, starch is known as an eco-friendly alternative material and offers the most beneficial prospect for continuous use [32]. This is because of its availability from renewable sources, low cost, and wide application possibilities in non-food and food products [32,46]. Generally, carbohydrate, the main stored substance in starch exists naturally as granules [59]. These granules are commonly obtained in stems, tubers, seeds, and leaves with diameters range between 1 µm to more than 100 µm with various shapes, e.g., polygonal, polyhedral, irregular, spherical, oval, and angular [60,61].

Up to now, several studies have been carried out on various parts of *Dioscorea hispida* plants. Tuber parts have been particularly identified and some of the recent research works published are tabulated in Table 1. It is evident from Table 1 that farming *Dioscorea hispida* plants can also generate large quantities of sustainable lignocellulosic materials every year, which can be used for the production of starch, bioplastic, and potential biocomposites to minimize environmental impact and renewability.

According to the literature, limited studies have been conducted on the investigation of the native starch and fibre solid wastes from *Dioscorea hispida* species as filler materials. In this study, the chemical, physical, morphological, and thermal properties of the *Dioscorea hispida* fibre and *Dioscorea hispida* starch were examined by Chemical Composition Investigation, Scanning Electron Microscopy (SEM), Particle Size Distribution, Thermogravimetric analysis (TGA), X-ray Powder Diffraction (XRD), and Fourier Transform Infrared (FTIR). To the best of our knowledge, no previous study had proposed *Dioscorea hispida* as a promising material with strong potential in the development of biodegradable films. In comparison with other natural fibres and starch, the objective of this work was to characterize novel starch and fibre from *Dioscorea hispida* tuber as renewable materials for food packaging applications. It must be noted that the *Dioscorea hispida* tubers used in this study were chemically treated to remove the toxin.

## 2. Materials and Methods

### 2.1. Materials

The native *Dioscorea hispida* was collected from a local farm M Abd Halim Enterprise in Kuala Terengganu, Malaysia. *Dioscorea hispida* starch and fibres were extracted from the fresh tubers. All samples were characterized in powder form.

### 2.2. Extraction of Dioscorea hispida Starch and Fibres

**Figure 1:**
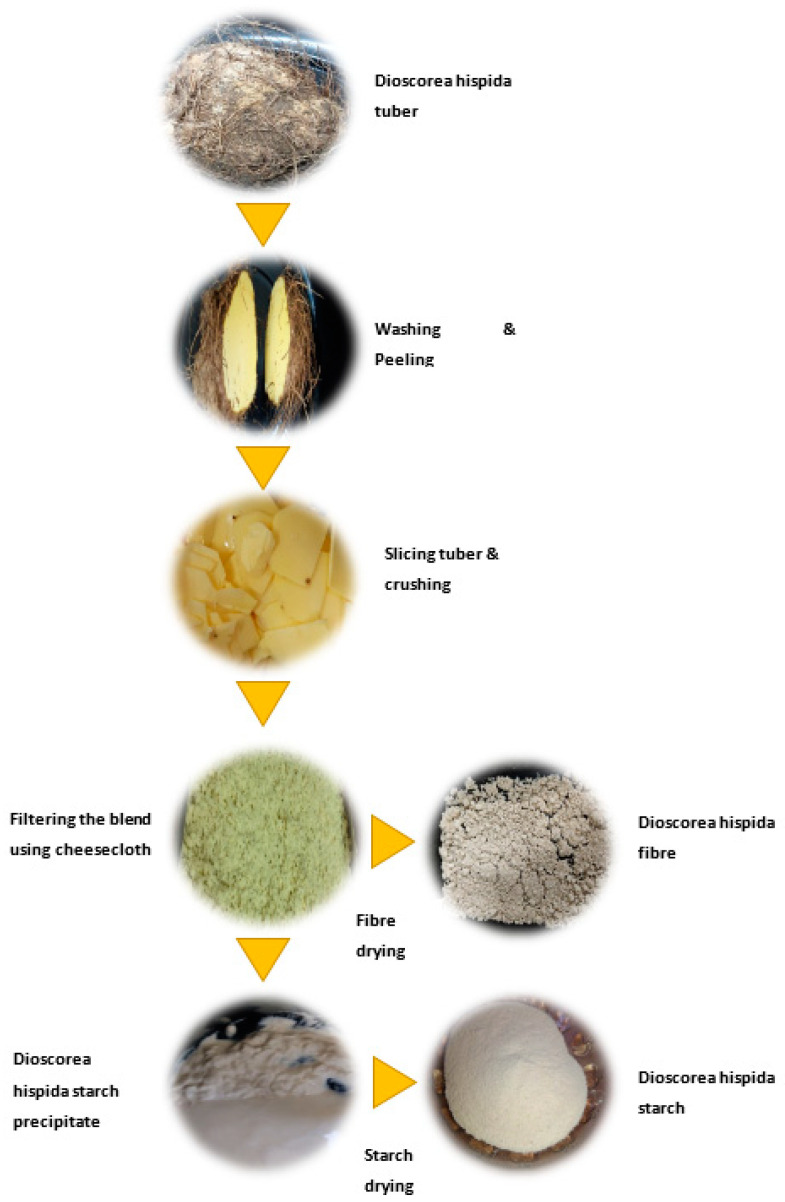
Extraction of *Dioscorea hispida* starch (DHS) and *Dioscorea hispida* fibres (DHF).

The extraction of *Dioscorea hispida* starch and fibre from the *Dioscorea hispida* tuber was conducted with a number of processes (Figure 1). Initially, the tubers were washed, peeled, and sliced [56]. Then, the slices were mixed with distilled water and crushed in a lab blender (HR2115/02, Phillips, Selangor, Malaysia) to obtain the minimum size. Later, the blended fractions were filtered through a cheesecloth. Finally, the white starch precipitate was separated and sun-dried. Meanwhile, the *Dioscorea hispida* fibres were obtained using a similar method but dried in an oven (Venticell 22, Planegg, Germany) at a temperature of 60 °C. The fibres were shredded, sieved through a 300 µm mesh sieve (Matest A060-01, MATEST S.p.A, Ancore, Italy) and characterized prior to application as filler in the *Dioscorea hispida* starch biocomposites.

### 2.3. Chemical Composition Analysis

The analysis was conducted to investigate the moisture, ash, starch, crude protein, energy, and carbohydrate contents of *Dioscorea hispida* starch. The procedures used to study acid detergent fibre (ADF), neutral detergent fibre (NDF), ash, lignin (LIG), hemicellulose, and cellulose of *Dioscorea hispida* fibres were adapted from [41]. The ADF and NDF were used in the determination of the chemical composition of *Dioscorea hispida* fibres. The most practised procedure was used for evaluating the amount of fibre constituents, such as hemicellulose, cellulose, and lignin. The amount of hemicellulose and cellulose were determined by using Equations (1) and (2), respectively.

Cellulose = ADF − lignin(1)

Hemicelluloses = NDF − ADF(2)

### 2.4. Density

The density was determined using an AccuPyc II 1340 pycnometer gas (Micromeritics Instrument Corp., Norcross, GA, USA) intrusion using helium gas flow. The samples underwent oven drying at a temperature of 105 °C for 24 h to eliminate the moisture content within the fibres and starch. Then, the samples were placed inside a desiccator to remove water traces from the dried sample before putting inside the pycnometer. Samples’ densities were calculated from measurements at a temperature of 27 °C using Equation (3).

(3) $$\rho =\frac{m}{V}$$

where, *m* = mass (g), *V* = volume (cm^3^)

### 2.5. Moisture Content

The moisture content investigation was performed with fibres and starch samples. The samples were heated in an oven at 105 °C for 24 h. The samples were weighed before, W_i_ and after, W_f_ the heating to evaluate the moisture content using Equation (4)

MC (%) = ((W_i_ − W_f_)/W_i_) × 100 (4)

### 2.6. Particle Size Distribution (PSD)

The instrument Mastersizer 2000 E Ver. 5.52 (Malvern Instruments Ltd., Worcestershire, UK) was used to identify the particle size distribution for all samples through a built-in Q-space powder feeder. Prior to distribution analysis, the particle size for the tested samples was investigated with a 1000 μm sieve.

### 2.7. Scanning Electron Microscopy (SEM)

The instrument scanning electron microscope (Hitachi S-3400N, Nara, Japan) was used to determine the samples’ surface morphology. The samples were coated with a layer of gold and 20 kV voltage was passed through at high vacuum condition to produce an electron beam. The electrons were connected with the sample atoms and produced signals giving a report on the surface topography, by generating images of high resolution.

### 2.8. Thermogravimetric Analysis (TGA)

The thermogravimetric analyser (Q500 V20.13, Build 39, Bellingham, WA, USA) was applied to analyse the thermal behaviour of the samples. The samples with the range of mass 5–10 mg were placed in platinum crucibles and the temperature was raised to 600 °C from room temperature under a heating rate of 10 °C/min in a nitrogen atmosphere with flow rate, 50 mL/min.

### 2.9. Fourier Transform Infrared Spectroscopy (FTIR)

The infrared spectrometer (Bruker Vector 22, Lancashire, UK) was used to obtain the FTIR spectrum for samples at frequencies over a wide spectral range, 4000 cm^−1^ to 400 cm^−1^ with 4 cm^−1^ of spectral resolution. The sample preparation was conducted using the potassium bromide (KBr) disc method, and the scan per specimen used was 16 scans.

### 2.10. X-ray Diffraction (XRD)

XRD analysis was conducted using a 2500 X-ray diffractometer (Instrument-Rigaku, Tokyo, Japan) with an angular range from 5° to 60° (2*θ*) at scattering speed of 0.02 (*θ*) s^−1^. The operating current and voltage were set to 35 mA and 40 kV, respectively.

## 3. Results

### 3.1. Chemical Composition

Table 2 compares the chemical composition between *Dioscorea hispida* starch and other natural starches. There were notable differences observed between the starch samples with regards to the ash, crude fat, crude protein, and starch amounts. Starch is widely found in the seed, root, and tubers of plants [68]. However, the chemical composition investigation revealed that the *Dioscorea hispida* starch (DHS) and starch had low moisture contents of 9.45% and 37.62%, respectively compared to cassava, corn, sugar palm, and arrowroot starches. Meanwhile, the *Dioscorea hispida* fibres (DHF) were significantly low in hemicellulose (4.36%), cellulose (5.63%), and lignin (2.79%) compared to cassava, corn hull, and sugar palm. The composition of these components could affect the characteristics of thermoplastic starch-based film composites, including the water barrier properties [69].

In addition, the ash content in the *Dioscorea hispida* starch was relatively high due to the presence of phosphate groups. In terms of metals, the ash of native starches consists mainly of potassium, calcium, sodium, and magnesium [72]. Carbohydrates content was more than 80% in *Dioscorea hispida* tuber composition, even though they differed according to genetic, ecological, and agronomic factors [39,67]. This was represented in the starch obtained from the chemical composition analysis, which had a carbohydrate content of 83% that was positively similar to the value provided in previous studies [73]. Since the aim was to extract the starch, high carbohydrate values were extremely significant.

The most important factor in the materials selection process is the molecular weight of the materials since it could affect the performance of the products [6]. Moreover, the density is the main criterion that correlates directly to this property. *Dioscorea hispida* starch showed slightly higher density, 1.74 g/cm^3^ compared to other natural starches shown in Table 2, respectively. The density value decreased due to the increase in volume with loss in weight of the starch.

The critical finding for *Dioscorea hispida* fibre is the amount of cellulose, hemicellulose, and lignin in the fibre. The results of this study were compared to other studies’ findings, as tabulated in Table 3. This increasing trend could be ascribed to the main components of the fibres, e.g., hemicellulose, and lignin. Moreover, it was shown that the hemicellulose (4.36%) and cellulose (5.63%) contents in *Dioscorea hispida* fibre were considerably low compared to other fibres. Furthermore, the lignin content (2.79%) in the *Dioscorea hispida* fibre was comparatively lower than the other samples’ lignin contents, as shown in Table 3. This amount was slightly lower by 57%, as previously discovered by Hamid et al. [53]. This result might be associated with the method of extraction for removing the toxin in *Dioscorea hispida* tubers. Apart from that, *Dioscorea hispida* fibre density (1.47 g/cm^3^**)** values were smaller compared to sugar palm fibres but higher than cassava and corn hull fibres [61,70].

Ibrahim et al. [61] reported that the lignin content was measured to determine the proportion of resistant components in the fibrous residue that performed a major function in producing strength to the fibre walls, as well as flexibility, and stiffness. The cellulose content in *Dioscorea hispida* fibres was relatively low compared to the other samples. The current study found that the relative amounts of different compounds discovered in natural fibres varied with each plant, including different parts of the same plant [60]. Generally, the mechanical strength of the natural fibres increased with the rising cellulose content of the fibres [77]. Previously published studies on the investigation of the natural fibres and their polymer composites have shown that the holocellulose contents consisting of hemicellulose and cellulose in natural fibres constituted 60–80% of the fibres, while the lignin composition was below 20% [78]. Similar results were also found for other natural fibre biomass wastes, e.g., the leaf, seed, and leaf stem [39,47].

Fibres are assumed to be natural source composites mainly consisting of holocellulose (hemicellulose, cellulose) and lignin, with low sugar, starch, protein, extractives, and ash content [46]. The incorporation of high cellulose quantities that allowed better matrix interactions, led to increased composite tensile strengths [32]. The mechanical properties were greatly improved when natural fibres were blended with thermoplastic starch and its blends [77]. The chemical similarity between starch and fibres was related to this fact, offering suitable composite compatibility [60]. Many studies found that the optimal performance of biodegradable starch/fibre film composites provided by corn starch and glycerol plasticizers was 10% lignin content [29,61]. The inconsistency in fibres might be attributed to the various compositions or origins of lignin and the various bioplastic preparation methods [79,80,81,82].

### 3.2. Thermogravimetric Analysis (TGA)

**Figure 2:**
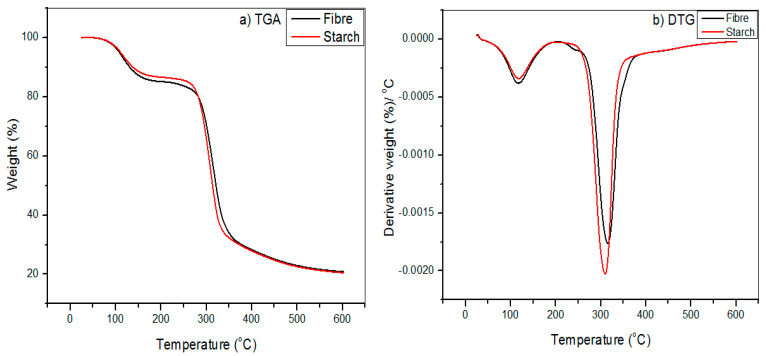
(**a**) TG and (**b**) DTG curves for *Dioscorea hispida* starch and *Dioscorea hispida* fibres.

Thermal stability is frequently assessed by identifying the thermal decomposition’s onset temperature. Figure 2 displays relative thermal stability values of the *Dioscorea hispida* starch and *Dioscorea hispida* fibre samples. It was apparent that considerable weight loss occurred up to ~100 °C in all of the samples as shown in Table 4. Increasing the temperature (>100 °C) resulted in a significant increase in the weight loss over a small temperature range as indicated by the lower slopes. However, the onset temperatures when weight loss started were similar to *Dioscorea hispida* starch and *Dioscorea hispida* fibre. For example, *Dioscorea hispida* starch sample’s onset thermal degradation temperature was about ~41.3 °C, while for samples of *Dioscorea hispida* fibre was ~40.7 °C. It might be due to the amount of moisture content of the samples (Table 2 and Table 3). The evaporation of the moisture from the *Dioscorea hispida fibre* was completed at 117.5 °C, which was lower compared to the *Dioscorea hispida* starch of 118.1 °C. This was due to the higher moisture content of *Dioscorea hispida* starch, which resulted in a greater mass loss (14.9%) than *Dioscorea hispida* fibre, as presented in Table 3. Visibly, Figure 2 shows that the earliest decomposition took place at a temperature lower than 100 °C, due to the evaporation of water or moisture [74]. Initially, as starch and fibres were heated, a reduction in the weight of the materials was noticed. This was due to the water and volatiles loss, which moved to the surface of the *Dioscorea hispida* starch and *Dioscorea hispida* fibre. The migration of volatile materials occurred as a result of the movement of water from the lower interior part of the starch and fibres to a better water potential region, at the surfaces of *Dioscorea hispida* starch and *Dioscorea hispida* fibre, as the water molecules on the surface of samples were evaporated. Hence, this movement of water indirectly moved the volatile materials, leaving them on the *Dioscorea hispida* starch and *Dioscorea hispida* fibre surfaces [32].

Nevertheless, the temperatures of the first thermal decomposition were about 260.4 °C for onset temperature of *Dioscorea hispida* starch and 203.9 °C for *Dioscorea hispida* fibre. The results showed that the initial heat tolerance of *Dioscorea hispida* starch was considerably greater than *Dioscorea hispida* fibre. It was revealed that when temperatures reached 140 °C, the inorganic materials and lignin of *Dioscorea hispida* fibre were degraded after the thermal decomposition of hemicellulose, cellulose, and volatiles [74]. The first phase of the *Dioscorea hispida* starch began with the decay of the water-soluble amylopectin at the onset temperature and continued until achieving a weight loss of approximately 66.1%. The highest rate of thermal degradation of *Dioscorea hispida* starch occurred at about 310 °C and left 20.6% residue of ash. These results were in good agreement with the findings in previous works [61,70].

When the temperatures were increased in the range of 200–300 °C, the observed degradation of *Dioscorea hispida* fibre was consistent with the degradation of hemicelluloses. The remarkable weight loss was due to the degradation of significant elements of lignocellulose, including hemicellulose, cellulose, and finally, lignin, which normally degrades at the temperature of higher than 140 °C [32]. Remarkably, when the temperature was raised from 350 °C to 550 °C, the degradation took place because of the presence of cellulose. It was ascribed with the dissipation of non-combustible gases, e.g., carbon monoxide and carbon dioxide that were present in the DHF samples. However, the devolatilization of these three components was seen to intersect, hence, these elements were considered as pseudo-components in *Dioscorea hispida* fibre, similar to other lignocellulosic material decompositions [23,74]. In addition, the residual mass of the *Dioscorea hispida* starch and *Dioscorea hispida* fibre samples were found to be in the range of 20% to 25% at a higher temperature (550 °C) (Table 2 and Table 3). This was possibly contributed to by the carbonaceous leftover in the nitrogen atmosphere, and a similar residue amount was reported in previous work [40]. Furthermore, several studies indicated that the wide decomposition temperature range was reported in other works and some researchers who studied the combustion kinetics of natural source materials demonstrated that the lignocellulose materials decompose between 300 °C to 350 °C [75,83]. Recent studies have revealed that lignin decomposition occurred over a wide temperatures range up to 900 °C [84]. The DTG curve of raw *Dioscorea hispida* starch and *Dioscorea hispida* fibre exhibited an earlier weight loss starting at 41.3 °C and 40.7 °C. The *Dioscorea hispida* fibre reached its highest peak at 315.4 °C because of the low hemicellulose and lignin decomposition temperature [6]. Hemicellulose was easily eliminated from the mainstream and consequently degraded to CO, CO_2_, and hydrocarbons at a low-temperature range, between 200 °C and 315 °C. As a result, the hemicellulose composition composed of different saccharides, e.g., galactose, mannose, xylose, glucose, and amorphous structure showed up as random and full of branches [85]. These results were consistent with the results obtained from the chemical composition investigations, FTIR and XRD.

### 3.3. Morphology and Particle Size Analysis

**Figure 3:**
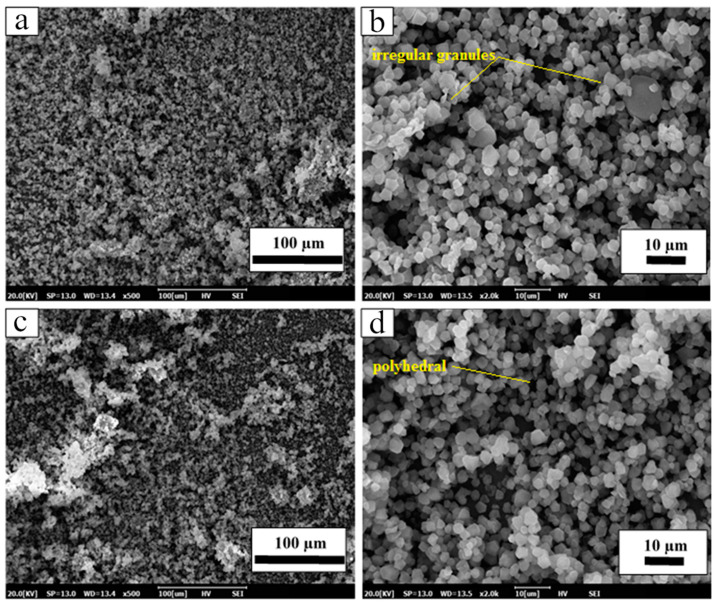
Scanning electron microscopy of magnification (**a**) DHF-500×, (**b**) DHF-2000×, (**c**) DHS-500×, (**d**) DHS-2000×.

SEM images of *Dioscorea hispida* starch and *Dioscorea hispida* fibre samples are displayed in Figure 3. The *Dioscorea hispida* starch appeared as a polyhedral shape obtained from the raw starch isolated from the *Dioscorea hispida* tubers with a range of size from 1.8 µm to 3.5 µm. The shape was different from other *Dioscorea* sp. due to the dissimilarity in biological sources and the environments of plant growth [86]. Previous studies reported comparable shapes of the *Dioscorea* sp. starch and found that *Dioscorea alata* had three different shapes; triangular, ellipsoid, and polyhedral [87]. In addition, other studies explored the irregular shapes of *Dioscorea opposita* starch such as cake-shaped, oval, and ground granules. Jiang et al. [88] published a paper describing the morphology of native starches relying on the biosynthesis of the starch granules, including several enzymes such as starch synthase, starch debranching enzyme, ADP-glucose pyro-phosphorylase, and starch branching enzyme together with the physiology of the plant-like amylose content, light transmittance percentage, swelling power, and water holding capacity [88]. The *Dioscorea hispida* fibre sample showed the presence of a polyhedral shape and exhibited individualized irregular granules with a clear smooth surface and no evidence of any fissure. The fibres were much bigger than *Dioscorea hispida* starch granules in this study with a range of size from 3.1 µm to 5.1 µm.

**Figure 4:**
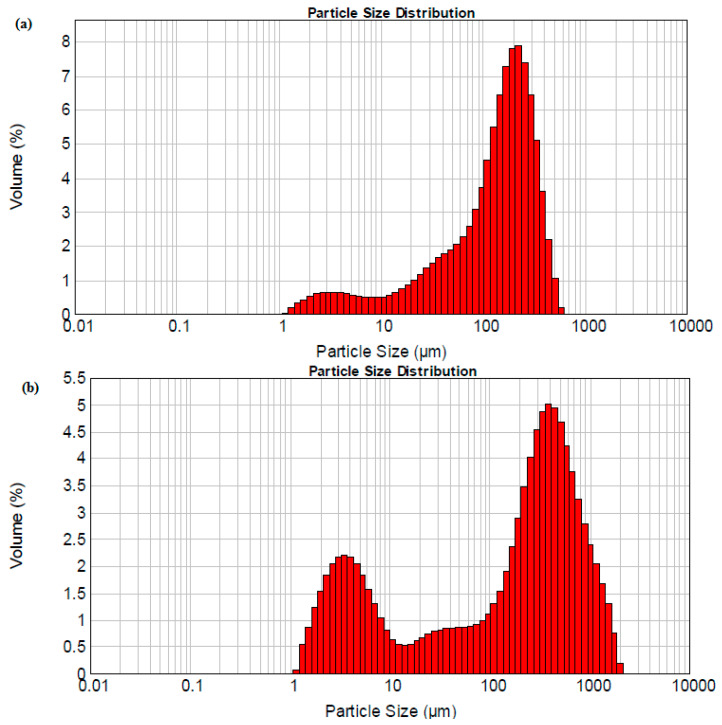
Particle size distribution of (**a**) *Dioscorea hispida* starch and (**b**) *Dioscorea hispida* fibre.

The particle size distribution of *Dioscorea hispida* samples showed a single mode distribution profile for their sizes. The graph was analyzed to approximate the gradation size range of the *Dioscorea hispida* fibre and *Dioscorea hispida* starch particles. According to the refractive guideline for the sample taken from the Malvern Instrument Mastersizer 2000, the refractive index value was denoted as 1.334. Thus, the particle size distribution of *Dioscorea hispida* fibre and *Dioscorea hispida* starch are presented in Figure 4. The graph showed that 10% of *Dioscorea hispida* fibre particles had dimensions of less than 18 µm and the majority sizes of the particles were 333 µm. Meanwhile, 10% of *Dioscorea hispida* starch particles had less than 3 µm and 877 µm for the majority sizes of the particles. However, the findings of the current study were unparalleled with the previous research of corn starch [29,61]. These results were likely to be related to the chemical composition properties of the materials.

The results indicated that the mean particle size of starch powder for the volume-weighted mean was 167 µm. The mean particle diameter demonstrated crystallinity pattern and degree of hardness inside the starch granules [89]. Recent evidence indicated that the higher particle size in native starch provided greater resistance of starch granules to mechanical force because of the intermolecular and intramolecular hydrogen bonding forces and the formation of a double helix between the amylopectin and amylose chains molecules [87].

Moreover, the particle size distribution of the fibres indicated that the volume-weighted mean was 344 µm. The *Dioscorea hispida* fibre sample presented a larger particle size compared to *Dioscorea hispida* starch due to the morphological structure related to the samples. Nonetheless, the findings of this study strengthened the idea that it can convey an excellent dispersion behaviour that is significant in reinforcing composites.

### 3.4. FTIR Spectroscopy Analysis

**Figure 5:**
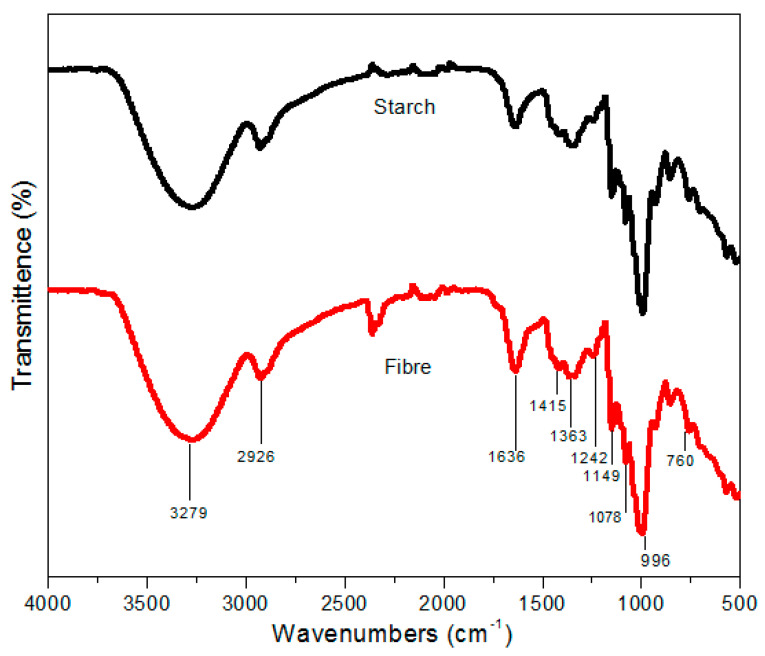
FTIR spectra of *Dioscorea hispida* starch and *Dioscorea hispida* fibre.

The FTIR spectra for raw *Dioscorea hispida* starch and fibre waste are portrayed in Figure 5. The curve of the FTIR spectra was divided into four main areas for interpretation of the analysis. The first stage occurred at wavenumbers below 800 cm^−1^, the second stage was between 800 cm^−1^ to 1500 cm^−1^, followed by the third stage within the C–H stretch area (2800 cm^−1^ to 3000 cm^−1^). The final stage showed intense peaks indicating the O–H groups’ presence in every filler part associated with the hydroxyl group discovered in hemicellulose, cellulose, and lignin [61]. Thus, the FTIR spectrum of the *Dioscorea hispida* starch and *Dioscorea hispida* fibre showed behaviour patterns below 800 cm^−1^ in the region related to the vibrations of the glucose pyranose unit [61]. The peak at 1149–1414 cm^−1^ in the FTIR spectrum of *Dioscorea hispida* fibre was mainly ascribed to the C=O stretching vibration of the ester linkage of the carboxylic group of ferulic and p-coumaric acids of hemicellulose and lignin [90]. It can be seen that both DHS and DHF roughly exhibited similar trends due to the differences related to the deviation in the samples’ chemical compositions. Furthermore, the peaks in the 1420–1410 cm^−1^ were shown as aromatic skeletal vibrations of the operative lignin group in DHF, while the band observed at 1635 cm^−1^ corresponded to lignin composition. These findings were supported by determining the chemical composition of *Dioscorea hispida* fibres as shown in Table 2. The peaks at 1414–1640 cm^−1^ indicated the structural polymer stretching of the aromatic groups existing in the lignin form. From Figure 4, the absorbance peaked between 1630–2930 cm^−1^ reflected the stretching of the C–H and O–H groups, correspondingly. Peaks found in the 3300–3000 cm^−1^ region were attributed to the adsorbed water. The absorbance bands around 856, 930, 996, 1077, 1149, and 1242 cm^−1^ were related to the C–O stretching, C–H rocking vibrations, and C–O–C asymmetric valence vibration, respectively, and these peaks indicated cellulose in the carbohydrates [91]. The *Dioscorea hispida* starch appeared to have complex spectra in the second region with outstanding overlapping originating from the monomer glucose units C–O vibrational stretching. This result might explain that the presence of the C–O–H bending mode was due to the band appearance at 1078 cm^−1^, while the coupling modes of C–C and C–O stretching exhibited the peak at 1148 cm^−1^. However, the peak at 1339 cm^−1^ of the infrared wavenumbers indicated CH_2_ bending modes [61]. In addition, the peak at 1638 cm^−1^ was related to the water fragment vibrations in the amorphous region of the starch granules, while another possible explanation for this was the emergence of broad infrared bands. The intense peaks at 3262 cm^−1^ meant the presence of O–H groups in *Dioscorea hispida* starch. A possible explanation for this might be the *Dioscorea hispida* starch that was very sensitive to water molecules due to the presence of hydroxyl groups. It can be noted that the fourth vibration in the region of the O–H stretch hydroxyl groups resulted in the formation of the last band [6].

### 3.5. X-ray Diffraction Analysis (XRD)

**Figure 6:**
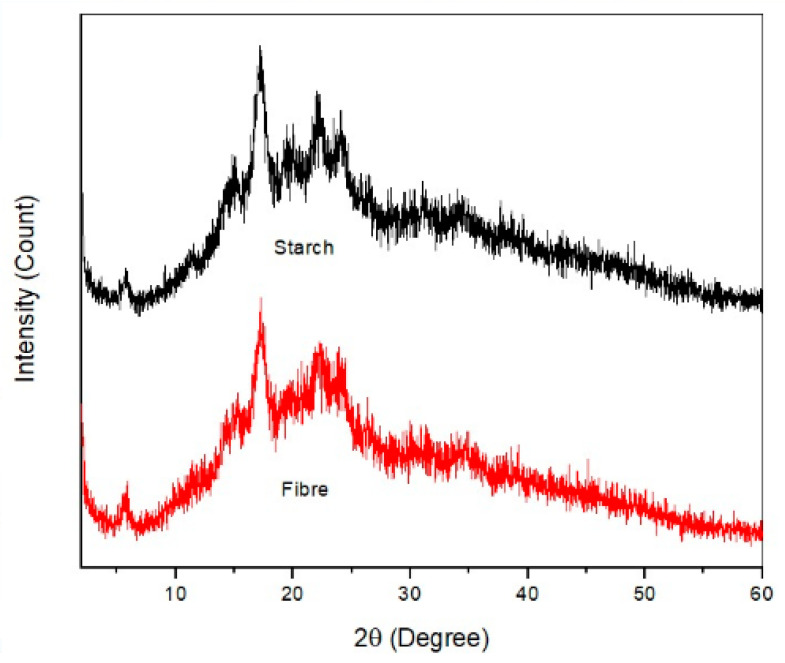
X-ray diffraction patterns of DHS and DHF.

XRD Analysis is frequently used to investigate the percentage of crystallinity and the structure as for the samples in this study. Figure 6 displays the XRD patterns for *Dioscorea hispida* starch and *Dioscorea hispida* fibre samples. The fibres characterization was performed via the XRD diffractograms. *Dioscorea hispida* fibres are primarily composed of cellulose, hemicellulose, and lignin, while the lignin is amorphous having a benzene ring with cellulose existing in a crystalline form in nature [88]. Based on Figure 6, the fibres and starch primarily indicated reflections at 2θ = 17.2° and 24.2° in the diffraction spectra, revealing that the forms of fibres and starch were almost similar, in terms of crystalline structure. However, the *Dioscorea hispida* starch possessed 27.5% crystallinity index, which was lower than *Dioscorea hispida* fibres of 39.0%. This was probably due to the difference in the samples’ contents, which was referred to the investigations of the chemical composition. The crystallinity index of *Dioscorea hispida* starch and *Dioscorea hispida* fibre supported the agreement of Ibrahim et al. [61] which revealed the crystallinity index of corn starch and fibres. Furthermore, the main crystalline peak at ~17.2° appeared equally as sharp for both *Dioscorea hispida* fibres and *Dioscorea hispida* starch samples. This might be attributed to the contents of lignin, cellulose, hemicellulose, moisture, and amylose of the fibres [6,74]. The highly crystalline nature of the samples contributed to their rigid structure which might favour the manufacture of thermoplastic starch composites, especially for food packaging application. Previous studies reported a higher amylose content of starch and a lower degree of crystallinity of starch. The results of this study were in keeping with previous observational studies of other groups of *Dioscorea* sp. which agreed on the crystallinity of the starches of *Dioscorea hispida* starch [87]. Therefore, the crystallinity value depends on the various types of plants and the preparation process to produce starch and fibres. It is noted that there is a relationship between the value of the crystallinity degree region and the properties of the materials, where the increment in the value of the crystallinity region increases the strength of the materials [6]. According to Han et al. [92], the modifications or treatment of starch and fibres are capable of destroying the crystalline region of materials by providing higher energy for the reaction between the granules and water molecules. Further investigations should be conducted to confirm this matter in the future.

## 4. Conclusions

*Dioscorea hispida* starch and *Dioscorea hispida* fibres were successfully extracted from *Dioscorea hispida* tubers after removing the dioscorine compound in the tubers. The chemical composition investigations, particle size distribution, scanning electron microscopy (SEM), X-ray powder diffraction (XRD), thermogravimetric analysis (TGA), and Fourier transform infrared (FT-IR) of DHS and DHF samples were explored, and their chemical composition, thermal properties, and morphologies were compared. The chemical composition investigations revealed that *Dioscorea hispida* starch has low moisture (9.45%) and starch content (37.62%) compared to cassava, corn, sugar palm, and arrowroot starches. Meanwhile, the *Dioscorea hispida* fibres (DHF) are significantly low in hemicellulose (4.36%), cellulose (5.63%), and lignin (2.79%) compared to cassava, corn hull, and sugar palm Thermogravimetric analysis (TGA) showed that the *Dioscorea hispida* starch maximum decomposition temperature was 309.7 °C, and for *Dioscorea hispida* fibres, it was 315.4 °C. The particle size determination and SEM analysis results also contributed to the excellent thermal stability of *Dioscorea hispida* starch and *Dioscorea hispida* fibres. The crystallinity index of *Dioscorea hispida* starch was 27.5%, which was lower than that of *Dioscorea hispida* fibres of 39.0%. This experiment revealed that *Dioscorea hispida* waste could be promising alternative biomass and sustainable material with excellent potential as a renewable filler material for food packaging applications. Tests with other geographical origins of *Dioscorea hispida* tubers should be conducted in a future study to determine the best characteristics for the production of biodegradable packaging films.

## Figures and Tables

**Table 1:** Previously published studies on *Dioscorea hispida* tuber biomass.

Parts of *Dioscorea hispida* Tubers	References
*Dioscorea hispida* tuber starch-polyacrylamide wood coating characterization	[52]
*Dioscorea hispida* tuber flour	[62]
A review on *Dioscorea hispida* tubers plant	[56]
Study on The Starch Granules Morphology	[63]
*Dioscorea hispida* as filler	[64]
Modified *Dioscorea hispida* starch	[58]
Tubers as a functional food	[65]
Distribution of *Dioscorea hispida*	[66]
*Dioscorea hispida* starch for edible coating	[67]
Chemical Composition of *Dioscorea hispida*	[53]

**Table 2:** Comparative chemical compositions of *Dioscorea hispida* starch/natural starch.

Natural Starch	Ash (%)	Crude Fat (%)	Crude Protein (%)	Moisture (%)	Starch (%)	Density (g/cm^3^)
*Dioscorea hispida*	2.33	0.02	5.55	9.45	37.62	1.74
Cassava [70]	0.31	-	0.56	12.66	58.82	1.48
Corn [61]	0.62	7.13	7.70	10.45	79.78	1.32
Sugar palm [30]	0.2	0.27	0.1	9.03	-	1.54
Arrowroot [71]	0.31	-	≈0	13.20	99.32	-

**Table 3:** Comparative chemical compositions of *Dioscorea hispida* fibre/natural fibre.

Natural Fibres	Cellulose (%)	Hemicellulose (%)	Lignin (%)	Ash (%)	Moisture (%)	Density (g/cm^3^)
*Dioscorea hispida*	5.63	4.36	2.79	1.28	9.15	1.47
Cassava [70]	10.04	29.26	3.12	3.36	14.92	1.45
Corn hull [61]	15.30	40.4	2.87	0.88	8.59	1.32
Sugar Palm Fibre [7]	43.88	7.24	33.24	1.01	8.36	1.50
Oil Palm Fibre [74]	43.70	29.02	13.33	3.31	-	-
Kenaf [75]	53.8	51.83	14.38	4	-	-
Sugarcane [76]	46.0	27.0	23.0	-	8.36	-

**Table 4:** Onset temperature (T_Onset_), thermal degradation on the maximum weight-loss rate (T_Max_), weight loss (W_L_) and char yield for *Dioscorea hispida* fibres and *Dioscorea hispida* starch obtained from the TG and DTG curves.

Samples	Water Evaporation			1st Thermal Degradation			2nd Thermal Degradation			Char Yield
	T_onset_ (°C)	T_max_ (°C)	W_L_ (%)	T_onset_ (°C)	T_max_ (°C)	W_L_ (%)	T_onset_ (°C)	T_max_ (°C)	W_L_ (%)	W(%)
DHS	41.3	118.1	14.9	260.4	309.7	66.1	-	-	-	20.6
DHF	40.7	117.5	13.4	203.9	240.5	4.3	255.1	315.4	64.3	20.8

## Data Availability

The data presented in this study are available on request from the corresponding author.

## References

[1] Ilyas R.A., Sapuan S.M., Ibrahim R., Abral H., Ishak M.R., Zainudin E.S., Atikah M.S.N., Mohd Nurazzi N., Atiqah A., Ansari M.N.M. (2019). Effect of sugar palm nanofibrillated celluloseconcentrations on morphological, mechanical andphysical properties of biodegradable films basedon agro-waste sugar palm (*Arenga pinnata (Wurmb.) Merr*) starch. J. Mater. Res. Technol..

[2] Alsubari S., Zuhri M.Y.M., Sapuan S.M., Ishak M.R., Ilyas R.A., Asyraf M.R.M. (2021). Potential of natural fiber reinforced polymer composites in sandwich structures: A review on its mechanical properties. Polymers.

[3] Sapuan S.M., Aulia H.S., Ilyas R.A., Atiqah A., Dele-Afolabi T.T., Nurazzi M.N., Supian A.B.M., Atikah M.S.N. (2020). Mechanical properties of longitudinal basalt/woven-glass-fiber-reinforced unsaturated polyester-resin hybrid composites. Polymers.

[4] Jumaidin R., Sapuan S.M., Jawaid M., Ishak M.R., Sahari J., Sapuan S.M., Jawaid M., Ishak M.R., Sahari J. (2017). Characteristics of *Eucheuma cottonii* waste from East Malaysia: Physical, thermal and chemical composition. Eur. J. Phycol..

[5] Ilyas R.A., Sapuan S.M., Ishak M.R., Zainudin E.S. (2019). Sugar palm nanofibrillated cellulose (*Arenga pinnata (Wurmb.) Merr*): Effect of cycles on their yield, physic-chemical, morphological and thermal behavior. Int. J. Biol. Macromol..

[6] Ilyas R.A., Sapuan S.M., Ishak M.R. (2018). Isolation and characterization of nanocrystalline cellulose from sugar palm fibres (Arenga Pinnata). Carbohydr. Polym..

[7] Ilyas R.A., Sapuan S.M., Ishak M.R., Zainudin E.S. (2017). Effect of delignification on the physical, thermal, chemical, and structural properties of sugar palm fibre. BioResources.

[8] Lisuzzo L., Cavallaro G., Milioto S., Lazzara G. (2020). Effects of halloysite content on the thermo-mechanical performances of composite bioplastics. Appl. Clay Sci..

[9] Syafri E., Sudirman, Mashadi, Yulianti E., Deswita, Asrofi M., Abral H., Sapuan S.M., Ilyas R.A., Fudholi A. (2019). Effect of sonication time on the thermal stability, moisture absorption, and biodegradation of water hyacinth (*Eichhornia crassipes*) nanocellulose-filled bengkuang (*Pachyrhizus erosus*) starch biocomposites. J. Mater. Res. Technol..

[10] Asrofi M., Sapuan S.M., Ilyas R.A., Ramesh M. (2020). Characteristic of composite bioplastics from tapioca starch and sugarcane bagasse fiber: Effect of time duration of ultrasonication (Bath-Type). Mater. Today Proc..

[11] Sanyang M., Sapuan S., Jawaid M., Ishak M., Sahari J. (2015). Effect of plasticizer type and concentration on tensile, thermal and barrier properties of biodegradable films based on sugar palm (*Arenga pinnata*) starch. Polymers.

[12] Ilyas R.A., Sapuan S.M., Ibrahim R., Abral H., Ishak M.R., Zainudin E.S., Atiqah A., Atikah M.S.N., Syafri E., Asrofi M. (2020). Thermal, biodegradability and water barrier properties of bio-nanocomposites based on plasticised sugar palm starch and nanofibrillated celluloses from sugar palm fibres. J. Biobased Mater. Bioenergy.

[13] Ilyas R.A., Sapuan S.M. (2020). Biopolymers and biocomposites: Chemistry and technology. Curr. Anal. Chem..

[14] Ilyas R.A., Sapuan S.M. (2020). The preparation methods and processing of natural fibre bio-polymer composites. Curr. Org. Synth..

[15] Jumaidin R., Khiruddin M.A.A., Asyul Sutan Saidi Z., Salit M.S., Ilyas R.A. (2020). Effect of cogon grass fibre on the thermal, mechanical and biodegradation properties of thermoplastic cassava starch biocomposite. Int. J. Biol. Macromol..

[16] Ilyas R.A., Sapuan S.M. (2019). Sugar palm (*Arenga pinnata [Wurmb.] Merr*) starch films containing sugar palm nanofibrillated cellulose as reinforcement: Water barrier properties. Polym. Compos..

[17] Omran A.A.B., Mohammed A.A.B.A., Sapuan S.M., Ilyas R.A., Asyraf M.R.M., Koloor S.S.R., Petrů M. (2021). Micro- and nanocellulose in polymer composite materials: A Review. Polymers.

[18] Rozilah A., Jaafar C.N.A., Sapuan S.M., Zainol I., Ilyas R.A. (2020). The effects of silver nanoparticles compositions on the mechanical, physiochemical, antibacterial, and morphology properties of sugar palm starch biocomposites for antibacterial coating. Polymers.

[19] Qin Y., Liu Y., Yong H., Liu J., Zhang X., Liu J. (2019). Preparation and characterization of active and intelligent packaging films based on cassava starch and anthocyanins from *Lycium ruthenicum Murr*. Int. J. Biol. Macromol..

[20] Singh G., Jose S., Kaur D., Soun B. (2020). Extraction and characterization of corn leaf fiber. J. Nat. Fibers.

[21] Pereira P.H.F., Souza N.F., Ornaghi H.L., de Freitas M.R. (2020). Comparative analysis of different chlorine-free extraction on oil palm mesocarp fiber. Ind. Crops Prod..

[22] Mali S., Debiagi F., Grossmann M.V.E., Yamashita F. (2010). Starch, sugarcane bagasse fibre, and polyvinyl alcohol effects on extruded foam properties: A mixture design approach. Ind. Crops Prod..

[23] Razali N., Salit M.S., Jawaid M., Ishak M.R., Lazim Y. (2015). A study on chemical composition, physical, tensile, morphological, and thermal properties of roselle fibre: Effect of fibre maturity. BioResources.

[24] Azammi A.M.N., Sapuan S.M., Ishak M.R., Sultan M.T.H. (2020). Physical and damping properties of kenaf fi bre fi lled natural rubber/thermoplastic polyurethane composites. Def. Technol..

[25] Chin S.C., Tee K.F., Tong F.S., Ong H.R., Gimbun J. (2020). Thermal and mechanical properties of bamboo fiber reinforced composites. Mater. Today Commun..

[26] Abral H., Ariksa J., Mahardika M., Handayani D., Aminah I., Sandrawati N., Sapuan S.M., Ilyas R.A. (2019). Highly transparent and antimicrobial PVA based bionanocomposites reinforced by ginger nanofiber. Polym. Test..

[27] Abral H., Ariksa J., Mahardika M., Handayani D., Aminah I., Sandrawati N., Pratama A.B., Fajri N., Sapuan S.M., Ilyas R.A. (2020). Transparent and antimicrobial cellulose film from ginger nanofiber. Food Hydrocoll..

[28] Acquavia M.A., Pascale R., Martelli G., Bondoni M., Bianco G. (2021). Natural Polymeric Materials: A Solution to Plastic Pollution from the Agro-Food Sector. Polymers.

[29] Hazrol M.D., Sapuan S.M., Zainudin E.S., Zuhri M.Y.M., Abdul Wahab N.I. (2021). Corn starch (zea mays) biopolymer plastic reaction in combination with sorbitol and glycerol. Polymers.

[30] Sanyang M.L., Sapuan S.M., Jawaid M., Ishak M.R., Sahari J. (2016). Recent developments in sugar palm (*Arenga pinnata*) based biocomposites and their potential industrial applications: A review. Renew. Sustain. Energy Rev..

[31] Abu Bakar M.S., Salit M.S., Mohamad Yusoff M.Z., Zainudin E.S., Ya H.H. (2020). The crashworthiness performance of stacking sequence on filament wound hybrid composite energy absorption tube subjected to quasi-static compression load. J. Mater. Res. Technol..

[32] Ilyas R.A., Sapuan S.M., Ishak M.R., Zainudin E.S. (2018). Development and characterization of sugar palm nanocrystalline cellulose reinforced sugar palm starch bionanocomposites. Carbohydr. Polym..

[33] Hazrol M.D., Sapuan S.M., Ilyas R.A., Othman M.L., Sherwani S.F.K. (2020). Electrical properties of sugar palm nanocrystalline cellulose reinforced sugar palm starch nanocomposites. Polimery.

[34] Aisyah H.A., Paridah M.T., Sapuan S.M., Ilyas R.A., Khalina A., Nurazzi N.M., Lee S.H., Lee C.H. (2021). A Comprehensive review on advanced sustainable woven natural fibre polymer composites. Polymers.

[35] Rangappa S.M., Siengchin S., Dhakal H.N. (2020). Green-composites: Ecofriendly and sustainability. Appl. Sci. Eng. Prog..

[36] Kumar T.S.M., Chandrasekar M., Senthilkumar K., Ilyas R.A., Sapuan S.M., Hariram N., Rajulu A.V., Rajini N., Siengchin S. (2020). Characterization, thermal and antimicrobial properties of hybrid cellulose nanocomposite films with in-situ generated copper nanoparticles in *Tamarindus indica* Nut Powder. J. Polym. Environ..

[37] Nurazzi N.M., Khalina A., Sapuan S.M., Ilyas R.A., Rafiqah S.A., Hanafee Z.M. (2020). Thermal properties of treated sugar palm yarn/glass fiber reinforced unsaturated polyester hybrid composites. J. Mater. Res. Technol..

[38] Sari N.H., Pruncu C.I., Sapuan S.M., Ilyas R.A., Catur A.D., Suteja S., Sutaryono Y.A., Pullen G. (2020). The effect of water immersion and fibre content on properties of corn husk fibres reinforced thermoset polyester composite. Polym. Test..

[39] Chandra Mohan C., Harini K., Vajiha Aafrin B., Lalitha priya U., Maria jenita P., Babuskin S., Karthikeyan S., Sudarshan K., Renuka V., Sukumar M. (2018). Extraction and characterization of polysaccharides from tamarind seeds, rice mill residue, okra waste and sugarcane bagasse for its bio-thermoplastic properties. Carbohydr. Polym..

[40] Lisuzzo L., Wicklein B., Lo Dico G., Lazzara G., del Real G., Aranda P., Ruiz-Hitzky E. (2020). Functional biohybrid materials based on halloysite, sepiolite and cellulose nanofibers for health applications. Dalt. Trans..

[41] Ilyas R.A., Sapuan S.M., Atikah M.S.N., Asyraf M.R.M., Rafiqah S.A., Aisyah H.A., Nurazzi N.M., Norrrahim M.N.F. (2021). Effect of hydrolysis time on the morphological, physical, chemical, and thermal behavior of sugar palm nanocrystalline cellulose (*Arenga pinnata (Wurmb.) Merr*). Text. Res. J..

[42] Asyraf M.R.M., Ishak M.R., Sapuan S.M., Yidris N., Ilyas R.A. (2020). Woods and composites cantilever beam: A comprehensive review of experimental and numerical creep methodologies. J. Mater. Res. Technol..

[43] Syafiq R., Sapuan S.M., Zuhri M.Y.M., Ilyas R.A., Nazrin A., Sherwani S.F.K., Khalina A. (2020). Antimicrobial activities of starch-based biopolymers and biocomposites incorporated with plant essential oils: A review. Polymers.

[44] Azammi A.M.N., Ilyas R.A., Sapuan S.M., Ibrahim R., Atikah M.S.N., Asrofi M., Atiqah A. (2019). Characterization studies of biopolymeric matrix and cellulose fibres based composites related to functionalized fibre-matrix interface. Interfaces in Particle and Fibre Reinforced Composites- From Macro to Nano Scales.

[45] Nazrin A., Sapuan S.M., Zuhri M.Y.M., Ilyas R.A., Syafiq R., Sherwani S.F.K. (2020). Nanocellulose reinforced thermoplastic starch (TPS), polylactic acid (PLA), and polybutylene succinate (PBS) for food packaging applications. Front. Chem..

[46] Jumaidin R., Sapuan S.M., Jawaid M., Ishak M.R., Sahari J. (2017). Thermal, mechanical, and physical properties of seaweed/sugar palm fibre reinforced thermoplastic sugar palm starch/agar hybrid composites. Int. J. Biol. Macromol..

[47] Nakthong N., Wongsagonsup R., Amornsakchai T. (2017). Characteristics and potential utilizations of starch from pineapple stem waste. Ind. Crops Prod..

[48] Mohd Izwan S., Sapuan S.M., Zuhri M.Y.M., Mohamed A.R. (2020). Effects of benzoyl treatment on naoh treated sugar palm fiber: Tensile, thermal, and morphological properties. J. Mater. Res. Technol..

[49] Ventura-Cruz S., Tecante A. (2019). Extraction and characterization of cellulose nanofibers from Rose stems (*Rosa* spp.). Carbohydr. Polym..

[50] Yan L., Chouw N., Jayaraman K. (2014). Flax fibre and its composites—A review. Compos. Part B Eng..

[51] Ganapathy T., Sathiskumar R., Senthamaraikannan P., Saravanakumar S.S., Khan A. (2019). Characterization of raw and alkali treated new natural cellulosic fibres extracted from the aerial roots of banyan tree. Int. J. Biol. Macromol..

[52] Lazim A.M., Azman I., Yusoff S.F.M., Hassan N.I., Fazry S., Arip M.N.M. (2016). Synthesis and characterization of Dioscorea hispida sp. tuber starch-polyacrylamide wood coating and its facile inhibitory towards *Pycnoporus sanguineus* and *Coptotermes curvignathus*. Prog. Org. Coatings.

[53] Hamid Z.A.A., Idris M.H.M., Arzami N.A.A.B., Ramle S.F.M. (2019). Investigation on the chemical composition of Discorea hispida dennst (Ubi Gadong). AIP Conf. Proc..

[54] Liu Y.W., Shang H.F., Wang C.K., Hsu F.L., Hou W.C. (2007). Immunomodulatory activity of dioscorin, the storage protein of yam (*Dioscorea alata cv.* Tainong No. 1) tuber. Food Chem. Toxicol..

[55] Sasiwatpaisit N., Thitikornpong W., Palanuvej C., Ruangrungsi N. (2014). Dioscorine content in Dioscorea hispida dried tubers in Thailand by TLC-densitometry and TLC image analysis. J. Chem. Pharm. Res..

[56] Kamaruddin Z.H., Sapuan S.M., Mohamed Yusoff M.Z., Jumaidin R. (2020). Rapid detection and identification of dioscorine compounds in dioscorea hispida tuber plants by LC-ESI-MS. BioResources.

[57] Nashriyah M., Salmah M.Y.T., Atiqah N., Indah O.S.N., MuhamadAzhar A.W., Munirah S., Nornasuha Y., Manaf A.A. (2012). Ethnobotany and distribution of *Dioscoreahispida dennst. (Dioscoreaceae)* in Terengganu, Peninsular Malaysia. World Acad. Sci. Eng. Technol..

[58] Ashri A., Amalina N., Kamil A., Fazry S., Sairi M.F., Nazar M.F., Lazim A.M. (2018). Modified Dioscorea hispida starch-based hydrogels and their in-vitro cytotoxicity study on small intestine cell line (FHS-74 Int). Int. J. Biol. Macromol..

[59] Pascoal A.M., Di-Medeiros M.C.B., Batista K.A., Leles M.I.G., Lião L.M., Fernandes K.F. (2013). Extraction and chemical characterization of starch from *S. lycocarpum* fruits. Carbohydr. Polym..

[60] Edhirej A., Sapuan S.M., Jawaid M., Zahari N.I. (2017). Preparation and characterization of cassava bagasse reinforced thermoplastic cassava starch. Fibers Polym..

[61] Ibrahim M.I.J., Sapuan S.M., Zainudin E.S., Zuhri M.Y.M. (2019). Extraction, chemical composition, and characterization of potential lignocellulosic biomasses and polymers from corn plant parts. BioResources.

[62] Kumoro A.C., Widiyanti M., Ratnawati R., Retnowati D.S. (2020). Nutritional and functional properties changes during facultative submerged fermentation of gadung (Dioscorea hispida Dennst) tuber flour using Lactobacillus plantarum. Heliyon.

[63] Mas S. (2016). Study on starch granules of local varieties of *Dioscorea hispida* and *dioscorea alata*. J. Trop. Life Sci..

[64] Susanto T., Affandy R., Katon G. (2018). Rahmaniar, Thermal aging properties of natural rubber-styrene butadiene rubber composites filled with modified starch from *Dioscorea Hispida Denst* extract prepared by latex compounding method. AIP Conf. Proc..

[65] Nugroho L.H., Estyaniyana A. (2018). The potency of gadung (*Dioscorea hispida Dennst.*) tuber as a functional food: Toxicity, phytochemical content and starch characters. AIP Conf. Proc..

[66] Mat N., Fatihah H.N.N., Sultan U., Abidin Z., Fatihah N., Nudin H., Arumugam N. (2014). The distribution of *dioscorea hispida Dennst.* Germplasm in Terengganu and phylogenetic relationships of *Dioscorea* spp. using Internal Transcribed Spacer (ITS). J. Agrobiotechnol..

[67] Alam P.N., Mukhlishien, Husin H., Asnawi T.M., Santia, Yustira A. (2019). The utilization of gadung (dioscorea hispida dennst) starch for edible coating making and its tomato packaging. IOP Conf. Ser. Mater. Sci. Eng..

[68] Baraheng S., Karrila T. (2019). Chemical and functional properties of durian (*Durio zibethinus Murr.*) seed flour and starch. Food Biosci..

[69] Ilyas R.A., Sapuan S.M., Ishak M.R., Zainudin E.S. (2018). Sugar palm nanocrystalline cellulose reinforced sugar palm starch composite: Degradation and water-barrier properties. IOP Conf. Ser. Mater. Sci. Eng..

[70] Edhirej A., Sapuan S.M., Jawaid M., Zahari N.I. (2017). Cassava: Its polymer, fiber, composite, and application. Polym. Compos..

[71] Andrés C., Gordillo S., Valencia G.A., Antonio R., Zapata V. (2014). Physicochemical characterization of arrowroot starch (*Maranta arundinacea Linn*) and glycerol/arrowroot starch membranes. Int. J. Food Eng..

[72] Swinkels J.J.M. (1985). Composition and properties of commercial native Starches. Starch Stärke.

[73] Polycarp D., Afoakwa E.O., Budu A.S., Otoo E. (2012). Characterization of chemical composition and anti-nutritional factors in seven species within the Ghanaian yam (*Dioscorea*) germplasm. Int. Food Res. J..

[74] Alotaibi M.D., Alshammari B.A., Saba N., Alothman O.Y., Sanjay M.R., Almutairi Z., Jawaid M. (2019). Characterization of natural fiber obtained from different parts of date palm tree (*Phoenix dactylifera* L.). Int. J. Biol. Macromol..

[75] Saba N., Abdan K., Ibrahim N.A. (2015). Preparation and characterization of fire retardant nano-filler from oil palm empty fruit bunch fibers. BioResources.

[76] Azlin M.N.M., Sapuan S.M., Zainudin E.S., Zuhri M.Y.M., Ilyas R.A., Al-Oqla F.M., Sapuan S.M. (2020). Natural polylactic acid-based fiber composites: A review. Advanced Processing, Properties, and Applications of Starch and Other Bio-Based Polymers.

[77] Kasim A.N., Selamat M.Z., Daud M.A.M., Yaakob M.Y., Putra A., Sivakumar D. (2016). Mechanical properties of polypropylene composites reinforced with alkaline treated pineapple leaf fibre from josapine cultivar. Int. J. Automot. Mech. Eng..

[78] Mendes C.A.D.C., Adnet F.A.D.O., Leite M.C.A.M., Furtado C.R.G., Sousa A.M.F. (2014). De Chemical, physical, mechanical, thermal and morphological characterization of corn husk residue. Cellul. Chem. Technol..

[79] Yang J., Ching Y., Chuah C. (2019). Applications of lignocellulosic fibers and lignin in bioplastics: A review. Polymers.

[80] Sabaruddin F.A., Paridah M.T., Sapuan S.M., Ilyas R.A., Lee S.H., Abdan K., Mazlan N., Roseley A.S.M., Abdul Khalil H.P.S. (2020). The effects of unbleached and bleached nanocellulose on the thermal and flammability of polypropylene-reinforced kenaf core hybrid polymer bionanocomposites. Polymers.

[81] Abral H., Chairani M.K., Rizki M.D., Mahardika M., Handayani D., Sugiarti E., Muslimin A.N., Sapuan S.M., Ilyas R.A. (2021). Characterization of compressed bacterial cellulose nanopaper film after exposure to dry and humid conditions. J. Mater. Res. Technol..

[82] Ayu R.S., Khalina A., Harmaen A.S., Zaman K., Isma T., Liu Q., Ilyas R.A., Lee C.H. (2020). Characterization study of empty fruit bunch (EFB) fibers reinforcement in Poly(Butylene) Succinate (PBS)/Starch/Glycerol Composite Sheet. Polymers.

[83] Galindez A., Daza L.D., Homez-Jara A., Eim V.S., Váquiro H.A. (2019). Characterization of ulluco starch and its potential for use in edible films prepared at low drying temperature. Carbohydr. Polym..

[84] Nasser R., Salem M., Hiziroglu S., Al-Mefarrej H., Mohareb A., Alam M., Aref I. (2016). Chemical analysis of different parts of date palm (*Phoenix dactylifera* L.) using ultimate, proximate and thermo-gravimetric techniques for energy production. Energies.

[85] Yang H., Yan R., Chen H., Lee D.H., Zheng C. (2007). Characteristics of hemicellulose, cellulose and lignin pyrolysis. Fuel.

[86] Ashri A., Yusof M.S.M., Jamil M.S., Abdullah A., Yusoff S.F.M., Nasir M.M. (2014). Physicochemical characterization of starch extracted from Malaysian wild yam (*Dioscorea hispida Dennst.*). Emir. J. Food Agric..

[87] Shujun W., Hongyan L., Wenyuan G., Haixia C., Jiugao Y., Peigen X. (2006). Characterization of new starches separated from different Chinese yam (*Dioscorea opposita Thunb.*) cultivars. Food Chem..

[88] Jiang Q., Gao W., Li X., Xia Y., Wang H., Wu S., Huang L., Liu C., Xiao P. (2012). Food hydrocolloids characterizations of starches isolated from five different *Dioscorea L.* species. Food Hydrocoll..

[89] Bhat F.M., Riar C.S. (2016). Effect of amylose, particle size & morphology on the functionality of starches of traditional rice cultivars. Int. J. Biol. Macromol..

[90] Mandal A., Chakrabarty D. (2011). Isolation of nanocellulose from waste sugarcane bagasse (SCB) and its characterization. Carbohydr. Polym..

[91] Alemdar A., Sain M. (2008). Isolation and characterization of nanofibers from agricultural residues - Wheat straw and soy hulls. Bioresour. Technol..

[92] Han Z., Zeng X.A., Fu N., Yu S.J., Chen X.D., Kennedy J.F. (2012). Effects of pulsed electric field treatments on some properties of tapioca starch. Carbohydr. Polym..

